# A second monoclinic polymorph of *N*-(pyrazin-2-yl)aniline

**DOI:** 10.1107/S1600536808031954

**Published:** 2008-10-11

**Authors:** Zanariah Abdullah, Seik Weng Ng

**Affiliations:** a Department of Chemistry, University of Malaya, 50603 Kuala Lumpur, Malaysia

## Abstract

The two aromatic rings in the title compound, C_10_H_9_N_3_, are aligned at 23.4 (1)° and the bridging C—N—C angle is  128.9 (1)°. In the crystal structure, intermolecular N—H⋯N hydrogen bonds result in a chain motif, the repeat distance of which is half the *b* axial length of 8.8851 (3) Å.

## Related literature

In the *P*2_1_/*c* modification, the aromatic rings are aligned at 15.2 (1)°, and the repeat distance of the helical chain is half the *b*-axial length of 7.8423 (3) Å; see: Wan Saffiee *et al.* (2008 ▶).
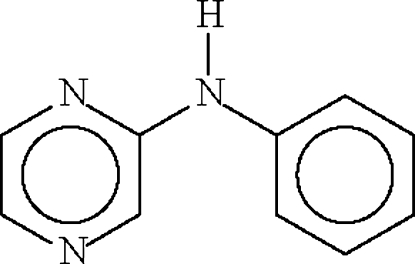


## Experimental

### 

#### Crystal data

C_10_H_9_N_3_
*M* *_r_* = 171.20
Monoclinic, 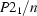

*a* = 8.2194 (3) Å
*b* = 8.8851 (3) Å
*c* = 11.8395 (4) Å
β = 104.643 (2)°
*V* = 836.56 (5) Å^3^
*Z* = 4
Mo *K*α radiation
μ = 0.09 mm^−1^
*T* = 100 (2) K
0.25 × 0.05 × 0.03 mm

#### Data collection

Bruker SMART APEX diffractometer
Absorption correction: none
7621 measured reflections
1922 independent reflections
1389 reflections with *I* > 2σ(*I*)
*R*_int_ = 0.045

#### Refinement

*R*[*F* ^2^ > 2σ(*F* ^2^)] = 0.042
*wR*(*F* ^2^) = 0.113
*S* = 1.03
1922 reflections
122 parameters
H atoms treated by a mixture of independent and constrained refinement
Δρ_max_ = 0.20 e Å^−3^
Δρ_min_ = −0.23 e Å^−3^

### 

Data collection: *APEX2* (Bruker, 2007 ▶); cell refinement: *SAINT* (Bruker, 2007 ▶); data reduction: *SAINT*; program(s) used to solve structure: *SHELXS97* (Sheldrick, 2008 ▶); program(s) used to refine structure: *SHELXL97* (Sheldrick, 2008 ▶); molecular graphics: *X-SEED* (Barbour, 2001 ▶); software used to prepare material for publication: *publCIF* (Westrip, 2008 ▶).

## Supplementary Material

Crystal structure: contains datablocks global, I. DOI: 10.1107/S1600536808031954/pk2124sup1.cif

Structure factors: contains datablocks I. DOI: 10.1107/S1600536808031954/pk2124Isup2.hkl

Additional supplementary materials:  crystallographic information; 3D view; checkCIF report

## Figures and Tables

**Table 1:** Hydrogen-bond geometry (Å, °)

*D*—H⋯*A*	*D*—H	H⋯*A*	*D*⋯*A*	*D*—H⋯*A*
N1—H1⋯N3^i^	0.90 (2)	2.17 (2)	3.062 (2)	175 (2)

Symmetry code: (i) 
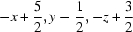
.

**Fig. 1.**
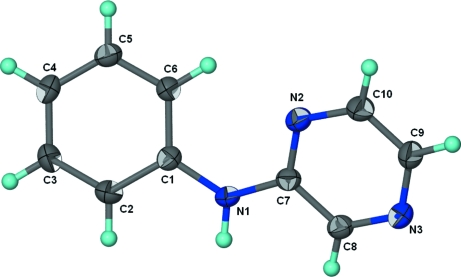
Thermal ellipsoid plot (Barbour, 2001) of C10H9N3 at the 70% probability level; hydrogen atoms are drawn as spheres of arbitrary radius.

## supplementary crystallographic information

### Comment

The cell dimensions of the reported monoclinic *P*2_1_/*c* modification are: a = 10.0644 (3), b = 7.8423 (3), c = 10.8907 (3) Å; β = 116.439 (2)° (Wan Saffiee *et al.*, 2008). The cell dimensions of the present modification (Scheme I, Fig. 1), after transformation to the standard *P*2_1_/*c* setting, are: a = 8.2194 (3), b = 8.8851 (3), c = 12.5909 (4) Å, β = 114.525 (2)°.

### Experimental

The *P*2_1_/*c* modification of 2-pyrazinyl-*N*-aniline (0.10 g, 0.4 mmol), zinc acetate (0.09 g, 0.4 mmol) and water (18 ml) were heated in a 23-ml Teflon-lined Parr bomb at 403 K for 2 days. The bomb was cooled to room temperature at 5 K min^-1^. Several faint yellow prisms were picked out manually from the cool solution.

### Refinement

Carbon-bound H-atoms were placed in calculated positions (C—H 0.95 Å) and were included in the refinement in the riding model approximation, with *U*(H) fixed at 1.2*U*(C). The amino H-atom was located in a difference Fourier map, and was freely refined.

### Crystal data

**Table:**

C_10_H_9_N_3_	*F*(000) = 360
*M**_r_* = 171.20	*D*_x_ = 1.359 Mg m^−^^3^
Monoclinic, *P*2_1_/*n*	Mo *K*α radiation, λ = 0.71073 Å
Hall symbol: -P 2yn	Cell parameters from 1282 reflections
*a* = 8.2194 (3) Å	θ = 2.7–26.1°
*b* = 8.8851 (3) Å	µ = 0.09 mm^−^^1^
*c* = 11.8395 (4) Å	*T* = 100 K
β = 104.643 (2)°	Prism, pale yellow
*V* = 836.56 (5) Å^3^	0.25 × 0.05 × 0.03 mm
*Z* = 4	

### Data collection

**Table:**

Bruker SMART APEX diffractometer	1389 reflections with *I* > 2σ(*I*)
Radiation source: fine-focus sealed tube	*R*_int_ = 0.045
graphite	θ_max_ = 27.5°, θ_min_ = 2.7°
ω scans	*h* = −10→10
7621 measured reflections	*k* = −11→11
1922 independent reflections	*l* = −15→14

### Refinement

**Table:**

Refinement on *F*^2^	Primary atom site location: structure-invariant direct methods
Least-squares matrix: full	Secondary atom site location: difference Fourier map
*R*[*F*^2^ > 2σ(*F*^2^)] = 0.042	Hydrogen site location: inferred from neighbouring sites
*wR*(*F*^2^) = 0.113	H atoms treated by a mixture of independent and constrained refinement
*S* = 1.03	*w* = 1/[σ^2^(*F*_o_^2^) + (0.0547*P*)^2^ + 0.1331*P*] where *P* = (*F*_o_^2^ + 2*F*_c_^2^)/3
1922 reflections	(Δ/σ)_max_ = 0.001
122 parameters	Δρ_max_ = 0.20 e Å^−^^3^
0 restraints	Δρ_min_ = −0.22 e Å^−^^3^

### Fractional atomic coordinates and isotropic or equivalent isotropic displacement parameters (Å^2^)

**Table:**

	*x*	*y*	*z*	*U*_iso_*/*U*_eq_	
N1	1.00115 (16)	0.42084 (14)	0.63143 (11)	0.0216 (3)	
H1	1.103 (2)	0.377 (2)	0.6527 (15)	0.035 (5)*	
N2	0.84988 (15)	0.63197 (14)	0.66840 (11)	0.0230 (3)	
N3	1.15770 (16)	0.76478 (14)	0.78284 (11)	0.0228 (3)	
C1	0.86787 (18)	0.33136 (16)	0.56827 (12)	0.0193 (3)	
C2	0.89595 (19)	0.17682 (17)	0.56499 (13)	0.0254 (4)	
H2	1.0021	0.1367	0.6049	0.030*	
C3	0.7717 (2)	0.08147 (17)	0.50462 (14)	0.0264 (4)	
H3	0.7933	−0.0235	0.5030	0.032*	
C4	0.61582 (19)	0.13750 (17)	0.44633 (13)	0.0232 (3)	
H4	0.5296	0.0717	0.4058	0.028*	
C5	0.58777 (19)	0.29106 (17)	0.44814 (13)	0.0222 (3)	
H5	0.4815	0.3305	0.4079	0.027*	
C6	0.71238 (18)	0.38836 (16)	0.50783 (13)	0.0211 (3)	
H6	0.6916	0.4936	0.5074	0.025*	
C7	0.99583 (18)	0.56191 (16)	0.67668 (13)	0.0192 (3)	
C8	1.14967 (18)	0.63040 (16)	0.73478 (13)	0.0213 (3)	
H8	1.2514	0.5772	0.7393	0.026*	
C9	1.00948 (18)	0.83515 (18)	0.77382 (13)	0.0248 (4)	
H9	1.0085	0.9325	0.8069	0.030*	
C10	0.8604 (2)	0.76892 (17)	0.71788 (14)	0.0254 (4)	
H10	0.7592	0.8226	0.7139	0.030*	

### Atomic displacement parameters (Å^2^)

**Table:**

	*U*^11^	*U*^22^	*U*^33^	*U*^12^	*U*^13^	*U*^23^
N1	0.0144 (7)	0.0200 (7)	0.0276 (7)	0.0019 (5)	0.0003 (5)	−0.0014 (5)
N2	0.0173 (6)	0.0230 (7)	0.0272 (7)	0.0004 (5)	0.0027 (5)	−0.0036 (5)
N3	0.0195 (7)	0.0228 (7)	0.0251 (7)	−0.0033 (5)	0.0037 (5)	−0.0011 (5)
C1	0.0180 (7)	0.0205 (7)	0.0193 (7)	−0.0010 (6)	0.0048 (6)	−0.0009 (6)
C2	0.0217 (8)	0.0227 (8)	0.0289 (8)	0.0040 (6)	0.0012 (6)	0.0000 (6)
C3	0.0293 (9)	0.0176 (8)	0.0307 (9)	0.0005 (6)	0.0043 (7)	−0.0019 (6)
C4	0.0217 (8)	0.0233 (8)	0.0244 (8)	−0.0059 (6)	0.0053 (6)	−0.0035 (6)
C5	0.0173 (7)	0.0251 (8)	0.0229 (8)	0.0005 (6)	0.0026 (6)	−0.0006 (6)
C6	0.0195 (8)	0.0190 (7)	0.0237 (8)	0.0005 (6)	0.0031 (6)	−0.0011 (6)
C7	0.0175 (7)	0.0198 (7)	0.0194 (7)	0.0004 (6)	0.0030 (6)	0.0020 (6)
C8	0.0177 (7)	0.0224 (8)	0.0233 (8)	−0.0001 (6)	0.0041 (6)	0.0015 (6)
C9	0.0228 (8)	0.0220 (8)	0.0284 (8)	−0.0016 (6)	0.0047 (6)	−0.0049 (6)
C10	0.0202 (8)	0.0241 (8)	0.0307 (9)	0.0032 (6)	0.0043 (6)	−0.0041 (7)

### Geometric parameters (Å, °)

**Table:**

N1—C7	1.3681 (19)	C3—H3	0.9500
N1—C1	1.4061 (19)	C4—C5	1.385 (2)
N1—H1	0.897 (18)	C4—H4	0.9500
N2—C7	1.3330 (18)	C5—C6	1.389 (2)
N2—C10	1.3438 (19)	C5—H5	0.9500
N3—C8	1.3171 (19)	C6—H6	0.9500
N3—C9	1.3494 (19)	C7—C8	1.415 (2)
C1—C6	1.393 (2)	C8—H8	0.9500
C1—C2	1.395 (2)	C9—C10	1.370 (2)
C2—C3	1.379 (2)	C9—H9	0.9500
C2—H2	0.9500	C10—H10	0.9500
C3—C4	1.385 (2)		
			
C7—N1—C1	128.94 (13)	C4—C5—H5	119.4
C7—N1—H1	114.0 (12)	C6—C5—H5	119.4
C1—N1—H1	116.7 (11)	C5—C6—C1	119.86 (14)
C7—N2—C10	115.67 (13)	C5—C6—H6	120.1
C8—N3—C9	116.12 (13)	C1—C6—H6	120.1
C6—C1—C2	118.72 (13)	N2—C7—N1	121.09 (13)
C6—C1—N1	123.92 (13)	N2—C7—C8	120.79 (13)
C2—C1—N1	117.35 (13)	N1—C7—C8	118.11 (13)
C3—C2—C1	120.88 (14)	N3—C8—C7	122.74 (13)
C3—C2—H2	119.6	N3—C8—H8	118.6
C1—C2—H2	119.6	C7—C8—H8	118.6
C2—C3—C4	120.52 (14)	N3—C9—C10	121.23 (14)
C2—C3—H3	119.7	N3—C9—H9	119.4
C4—C3—H3	119.7	C10—C9—H9	119.4
C5—C4—C3	118.88 (14)	N2—C10—C9	123.44 (14)
C5—C4—H4	120.6	N2—C10—H10	118.3
C3—C4—H4	120.6	C9—C10—H10	118.3
C4—C5—C6	121.11 (14)		
			
C7—N1—C1—C6	21.9 (2)	C10—N2—C7—N1	178.88 (14)
C7—N1—C1—C2	−159.27 (15)	C10—N2—C7—C8	0.2 (2)
C6—C1—C2—C3	−1.0 (2)	C1—N1—C7—N2	2.9 (2)
N1—C1—C2—C3	−179.81 (14)	C1—N1—C7—C8	−178.36 (14)
C1—C2—C3—C4	−0.3 (2)	C9—N3—C8—C7	0.0 (2)
C2—C3—C4—C5	1.0 (2)	N2—C7—C8—N3	−0.2 (2)
C3—C4—C5—C6	−0.5 (2)	N1—C7—C8—N3	−178.92 (13)
C4—C5—C6—C1	−0.8 (2)	C8—N3—C9—C10	0.1 (2)
C2—C1—C6—C5	1.5 (2)	C7—N2—C10—C9	0.0 (2)
N1—C1—C6—C5	−179.75 (13)	N3—C9—C10—N2	−0.1 (2)

### Hydrogen-bond geometry (Å, °)

**Table:**

*D*—H···*A*	*D*—H	H···*A*	*D*···*A*	*D*—H···*A*
N1—H1···N3^i^	0.90 (2)	2.17 (2)	3.062 (2)	175 (2)

Symmetry codes: (i) −*x*+5/2, *y*−1/2, −*z*+3/2.
